# Performance of automated respiratory rate counters for respiratory rate assessment to support pneumonia detection among children under five years of age in low- and middle-income settings: a systematic review of qualitative and quantitative evidence

**DOI:** 10.7189/jogh.16.04281

**Published:** 2026-08-21

**Authors:** Sifat Binte Ibrahim, Kazi Sazzadul Haque, M Z E M Naser Uddin Ahmed, Md Jasim Uddin, Salahuddin Ahmed, Saima Sultana, Ahad Mahmud Khan

**Affiliations:** 1 Projahnmo Research Foundation, Dhaka, Bangladesh; 2 Women's, Children's and Adolescents' Health Program, Burnet Institute, Melbourne, Australia

**Keywords:** pneumonia, respiratory rate, fast breathing, automated respiratory rate counter, IMCI, LMICs

## Abstract

**Background:**

Accurate respiratory rate (RR) assessment is essential for pneumonia identification under World Health Organization Integrated Management of Childhood Illness (IMCI) guidelines; however, manual RR counting is often inaccurate and difficult in routine care. Automated RR counters have been developed to address these challenges, but evidence on their diagnostic performance and implementation in low- and middle-income countries (LMICs) remains limited. This systematic review evaluated the accuracy, usability, acceptability and time efficiency of automated RR counters for RR assessment to support identifying pneumonia in children aged 0–59 months in LMICs.

**Methods:**

We searched MEDLINE, EMBASE, Web of Science, and Scopus for studies published between 1 January 2014 and 15 July 2025. Studies conducted in LMICs assessing automated RR counters in children aged 0–59 months were eligible, including quantitative, qualitative, and mixed-methods designs. Study quality was assessed using the Joanna Briggs Institute appraisal tools. Quantitative findings were synthesised narratively, and qualitative findings were analysed thematically.

**Results:**

Fourteen studies from seven LMICs were included. Two automated RR counters – Masimo Rad-G and ChARM – were evaluated. Masimo Rad-G showed variable performance across studies (sensitivity = 75.9%–95.4%; specificity = 93.8%–98.3%; kappa = 0.55–0.85, n = 3), while ChARM demonstrated high diagnostic performance (sensitivity = 95.8%; specificity = 93.5%; kappa = 0.74–0.86, n = 2). Usability studies reported generally positive user experiences, though performance was affected by child movement, adherence to measurement procedures, and device design. Health workers and caregivers perceived automated tools as more reliable than manual counting, with visual indicators improving confidence in clinical decisions. Reported challenges included battery life, maintenance, workflow disruption, and the need for continuous training and supervision.

**Conclusions:**

Although the available evidence remains limited, automated RR counters show promise for RR assessment in LMIC settings to support pneumonia identification. Future studies are needed to confirm their effectiveness and implementation feasibility across diverse routine-care settings. Successful adoption is likely to depend on adequate training, device optimisation, and integration into existing health-system workflows.

**Registration:**

PROSPERO: CRD420251080564

Pneumonia is a major cause of morbidity and mortality among children under five years of age worldwide, accounting for approximately 14% of deaths in this age group [1]. Despite substantial global progress in child survival, the burden of pneumonia continues to be disproportionately high in low- and middle-income countries (LMICs), where the vast majority of pneumonia-related deaths occur [2,3]. In these settings, limited diagnostic capacity, shortages of trained healthcare providers, and constrained health system resources contribute to delayed diagnosis and treatment [4,5].

Early and accurate detection of pneumonia is essential to reduce mortality and disease progression [6]. Within the World Health Organization (WHO) Integrated Management of Childhood Illness (IMCI) guidelines, respiratory rate (RR) is the central component for identifying fast breathing, a key clinical sign of pneumonia [7]. In routine practice, RR is traditionally measured manually over 60 seconds using either an acute respiratory illness (ARI) timer or a watch [8]. However, manual RR counting in children is inherently challenging and prone to error. Factors such as inadequate training, high patient workload, observer variability, or poor cooperation from restless or distressed children contribute to inconsistent and inaccurate measurements [9–15]. These may lead to misclassification of fast breathing and inappropriate treatment decisions, including unnecessary or delayed antibiotic use [16,17].

Recognising these challenges, the United Nations Children's Fund (UNICEF) launched the Acute Respiratory Infection Diagnostic Aids (ARIDA) initiative in 2014–2015 to promote the development of improved tools for RR assessment and pneumonia identification in children [18]. A target product profile (TPP) was subsequently developed to guide manufacturers and researchers in designing automated RR counters suitable for use in low-resource settings [19]. These devices are intended to measure RR automatically, display the result, and indicate whether the measured rate exceeds age-specific fast-breathing thresholds defined by the WHO IMCI guidelines [8].

Automated RR counters have the potential to address many limitations associated with manual counting by providing objective, standardised, and time-efficient RR measurements that can be used by frontline health workers [20]. However, despite increasing technological innovation, their implementation in LMIC settings remains limited. Many devices were initially developed for continuous physiological monitoring in well-resourced healthcare environments rather than for point-of-care use in primary healthcare or community settings [21,22]. As newer technologies continue to emerge, it is essential to assess whether these devices have been adequately evaluated under real-world conditions relevant to low-resource contexts.

Although several individual studies have evaluated automated RR counters in children under five years of age, the existing evidence remains fragmented, with variations in study design, reference standards, and outcome measures. To date, the evidence has not been systematically synthesised to provide a comprehensive understanding of device performance, usability, and operational feasibility in LMIC settings. Therefore, this systematic review aims to summarise and critically appraise the available evidence on the performance of automated RR counters in measuring RR and identifying fast breathing among children. In addition, this review examines usability, acceptability, time efficiency, and the potential impact of these technologies on improving RR measurement and clinical decision-making in LMICs.

## METHODS

We conducted this systematic review in accordance with the Preferred Reporting Items for Systematic Reviews and Meta-Analyses (PRISMA) 2020 guidelines [23]. The review protocol was prospectively registered in the International Prospective Register of Systematic Reviews (PROSPERO) (Registration ID: CRD420251080564; registered 25 June 2025) [24]. The methodological approach was further guided by recommendations outlined in the Cochrane Handbook for Systematic Reviews of Interventions [25].

### Search strategy

We conducted comprehensive literature search in the following electronic databases: MEDLINE (*via* Ovid), EMBASE (*via* Ovid), Web of Science, and Scopus, covering the period from 1 January 2014 to 15 July 2025. The search strategy was developed with methodological support for systematic reviews and incorporated both Medical Subject Headings (MeSH) and free-text terms related to pneumonia, RR measurement, automated RR counters, and children under five years of age. Boolean operators (‘AND’, ‘OR’) and truncation were applied to capture variations in terminology across databases. The reference lists of included studies and relevant systematic reviews were manually searched to identify additional eligible publications. Only studies published in English were included. The complete search strategy and database-specific search strings are provided in Table S1 in the **Online Supplementary Document**.

### Eligibility criteria

We selected studies based on predefined inclusion and exclusion criteria. Because the review aimed to evaluate both device performance and implementation-related outcomes, a broad range of study designs was considered initially.

#### Inclusion criteria

We included quantitative, qualitative, and mixed-methods research, including observational, diagnostic accuracy, and implementation studies, as well as case reports and case series. Eligible studies involved children younger than five years and evaluated automated or multimodal RR counters used at the point of care. Studies were required to report at least one relevant outcome, including device performance, usability, acceptability, time efficiency, or potential impact on the accuracy of RR measurement. We included only studies conducted in LMICs, as defined by the World Bank classification [26].

#### Exclusion criteria

We excluded review articles, editorials, study protocols, and commentaries. We also excluded studies that did not report separate data for children under five years of age, non-English publications, and studies conducted exclusively in high-income countries.

### Study selection

We imported all records identified through database searches into Covidence systematic review software [27], where automated duplicate removal was performed. Two reviewers (SBI and KSH) independently screened titles and abstracts against the predefined eligibility criteria. We excluded studies at this stage if they were clearly irrelevant, including those not related to pneumonia or RR measurement, not involving children under five years of age, not evaluating automated RR devices, or conducted exclusively in high-income settings. Where eligibility could not be determined from the title and abstract alone, studies were retained for full-text review to minimise the risk of premature exclusion. The same reviewers (SBI and KSH) independently assessed full texts of potentially eligible articles. Disagreements at either screening stage were resolved through discussion, and when consensus could not be reached, a third reviewer (MZEMNUA) adjudicated the decision.

### Data extraction

The reviewers independently conducted data extraction using a standardised and pre-piloted extraction form (Table S2 in the **Online Supplementary Document**). For all included studies, extracted information included study characteristics (author, year, journal, and country), study design and setting, participant characteristics (sample size, age range, and eligibility criteria), and device characteristics. For studies reporting quantitative outcomes, additional data were extracted on comparator or reference standard (*e.g.* video expert panel counts, physician counts, or health worker counts), and reported performance measures, including diagnostic accuracy and agreement outcomes where available. For studies reporting qualitative findings, relevant participant quotations and author reported findings related to the review objectives were extracted. In mixed-methods studies, quantitative and qualitative data were extracted separately according to their respective evidence domains and incorporated into the corresponding quantitative and qualitative syntheses. This approach facilitated separate synthesis of evidence streams and minimised inappropriate double-counting during interpretation.

### Data analysis and synthesis

We analysed quantitative and qualitative findings separately. Due to substantial heterogeneity in study designs, populations, outcome measures, reference standards, and reporting methods, meta-analysis was not considered appropriate. Quantitative findings were therefore synthesised narratively. Reported performance metrics, including sensitivity, specificity, agreement measures, and kappa statistics, were summarised descriptively where available.

We synthesised qualitative findings using thematic synthesis informed by Thomas and Harden [28]. Relevant quotes and author-mentioned themes were examined line-by-line and coded based on meaning. Both semantic coding (direct meaning) and latent coding (underlying meaning) were applied to capture participants’ experiences comprehensively, where supported by the available data. Coding was iterative, with new codes added and existing codes refined as additional studies were analysed. Codes were compared across studies and grouped into sub-themes based on conceptual similarity. These sub-themes were then compared and interpreted across studies to develop higher-order themes. The steps of thematic synthesis are provided in Figure S1 in the **Online Supplementary Document**. Because several included studies provided limited qualitative detail, the synthesis primarily generated descriptive themes related to the objectives of the study, while incorporating interpretive insights that were supported by the available data.

For mixed-methods studies, qualitative and quantitative findings contributed only to the respective synthesis and were analysed independently of their respective (qualitative/quantitative) components.

Quantitative and qualitative evidence streams remained linked to the parent study but were synthesised separately to minimise inappropriate double-counting and to preserve transparency in interpretation. Illustrative participant quotations were retained to support interpretation. The number of studies contributing to each theme was documented to enhance transparency.

### Risk of bias assessment

Two reviewers (SBI and KSH) assessed risk of bias independently by two reviewers (SBI and KSH) using the Joanna Briggs Institute (JBI) Critical Appraisal Checklists, selected according to study design [29]. Disagreements were resolved through discussion; unresolved cases were adjudicated by a third reviewer (MZEMNUA). The JBI Diagnostic Test Accuracy Checklist was used for studies evaluating device performance against a reference standard, the JBI Analytical Cross-Sectional Checklist for observational performance studies, and the JBI Checklist for Qualitative Research for studies assessing usability, acceptability, or feasibility.

For mixed-methods studies, we appraised quantitative and qualitative components separately using the checklist most appropriate for each component. This component-specific approach enabled independent assessment of methodological quality across different evidence domains while maintaining linkage to the parent study.

Checklist items were scored as 1 (‘yes’) or 0 (‘no’, ‘unclear’, or ‘not applicable’). Summary scores were calculated as a percentage of the maximum obtainable score for descriptive comparison across studies and presentation of appraisal results. These scores were not used to weight studies in the synthesis, determine study inclusion, or influence interpretation of findings.

## RESULTS

### Study selection

**Figure 1:**
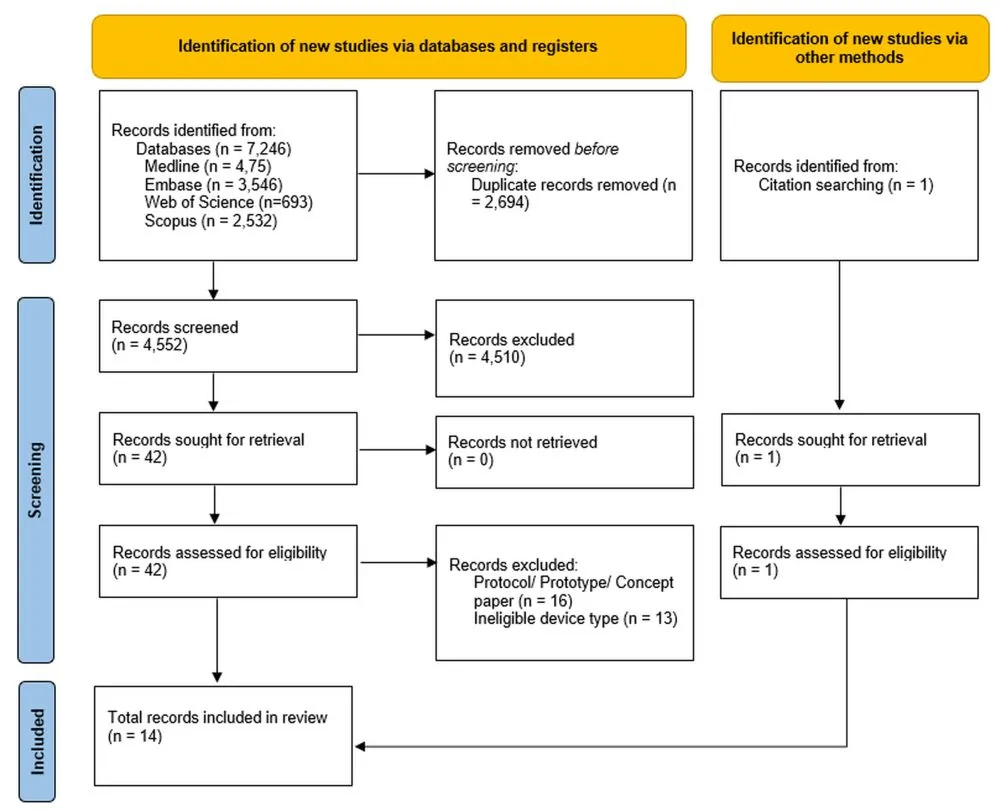
PRISMA flow diagram.

A total of 7,246 records were identified through database searches. After removal of 2,694 duplicates, 4,552 records remained for title and abstract screening, of which 4,510 were excluded. Forty-two articles were eligible for full text review. Of these, 29 were excluded for the following reasons: study protocols (n = 8), prototype devices without field evaluation (n = 8), and ineligible studies evaluating non-automated RR measurement devices (n = 13). One additional study was identified through citation searching. In total, 14 studies met the inclusion criteria and were included in the final review (**Figure 1**).

A detailed list of excluded studies, with reasons for exclusion, is provided in Table S3 of the **Online Supplementary Document**.

### Characteristics of the included studies

The characteristics of the included studies are presented in **Table 1**. The studies were conducted across seven LMICs: Ethiopia, Nepal, India, the Democratic Republic of Congo, Nigeria, Bangladesh, and Burundi. Of these, five were quantitative studies, five were qualitative studies, and four employed mixed methods designs. No case reports or case series met the inclusion criteria. Two automated RR counters- Masimo Rad-G and Children’s Automated Respiration Monitor (ChARM) were evaluated. The Masimo Rad-G was assessed in eight studies, while the ChARM device was evaluated in five studies; one study examined both devices.

**Table 1:** Characteristics of the included studies

SL	Author, Year	Country	Study Method	Setting	Participant	Sample Size	Sampling method	Device	Device used by	Number of HW	Reference Standard Details	Timing of reference standard	Outcome Measures
**1**	Ward C, 2019	Ethiopia	Mixed method	Community and Primary health facility	Under 5 children, HEWs, FLHFWs	Children: 599, 133 HEWs: 133, FLHFWs: 20	Convenience	ChARM	HEWs and FLHFWs	HEW: 133, FLHFW: 20	Not applicable	Not applicable	Usability, Acceptability
**2**	Källander K, 2020	Nepal	Mixed method	Community	0-59 months children, FCHVs	Children: 517	Convenience	ChARM	FCHVs	133	Not applicable	Not applicable	Usability, Acceptability
**3**	Alwadhi V, 2020	India	Quantitative	Hospital–outpatient and emergency	2 to 59 months children	Children: 97	Convenience	Masimo Rad-G	A study consultant	1	Manual RR count by paediatrician	Sequential	Diagnostic Test Accuracy/ Agreement
**4**	Baker K, 2020	Ethiopia	Mixed method	Community health posts and first-level health centres	Under 5 children, HEWs, FLHFWs	Child: 526, HEWs: 134, FLHFWs: 20	Convenience	Masimo Rad-G	HEWs and FLHFWs	HEWs: 134, FLHFWs: 20	Not applicable	Not applicable	Usability, Acceptability
**5**	Hellden D, 2020	Ethiopia	Quantitative	Hospital	2 to 59 months children	188	Consecutive	ChARM	Research nurse, Expert counter	Research nurse: 1, Expert counter: 1	Manual count by nurse	1 min, 3 min, and 5 min	Attachment Impact
**6**	Tack B, 2021	DR Congo	Quantitative	Hospital-inpatient	28 days to 59 months old children	202	Convenience	Masimo Rad-G	Trained research nurse	Not reported	Manual RR count by trained research nurse	Simultaneously	Diagnostic Test Accuracy, Agreement
**7**	Kumar H, 2021	India	Qualitative	Hospital-outpatient	Health care providers	23	Consecutive	Masimo Rad-G	Healthcare providers	Not reported	Not applicable	Not applicable	Usability, Acceptability
**8**	Sarin E, 2021	India	Qualitative	Hospital-outpatient	Health workers	11	Convenience	Masimo Rad-G	AYUSH medical officer, CHO, Staff Nurse and ANM	Total providers: 11	Not applicable	Not applicable	Usability, Acceptability
**9**	Dale N M, 2021	Nigeria	Quantitative	Hospital-inpatient	6-59 months old children with severe acute malnutrition	514	Convenience	Masimo Rad-G	Study nurses	5	Manual RR count by study nurses	Simultaneously	Agreement
**10**	Ghosh N, 2022	India	Mixed method	Community	Under 5 children, Caregivers, CHWs	132	Convenience	ChARM	ASHA and BPHN	ASHA: 17, BPHN: 2	Visual inspection and manual count by ASHA/BPHN	Sequential	Usability, Acceptability, Efficacy
**11**	Tack B, 2022	DR Congo	Qualitative	Hospital- triage	>28 days to <5-years-old children	Not reported	Convenience	Masimo Rad-G	Nurses, Physicians	Nurses: 6 and Physicians: 2	Not applicable	Not applicable	Usability
**12**	Rawat A, 2023	Ethiopia	Qualitative	Community and primary health facility	HW’s in communities and facilities, trainers of HW’s, district management, and key decision-makers	57	Convenience	Masimo Rad-G, ChARM	Not reported	Not reported	Not applicable	Not applicable	Consideration
**13**	Bauler S, 2025	Burundi	Qualitative	Community	CHWs	30	Convenience	Masimo Rad-G	CHW, Caregiver	CHWs: 30, Caregivers: 30	Not applicable	Not applicable	Usability, Acceptability, and Feasibility
**14**	Khan M, 2025	Bangladesh	Quantitative	Hospital	0 to 59 months children	339	Consecutive	ChARM	Physician, CHCP	Not reported	VEP of physicians with device recording.	Simultaneously	Diagnostic Test Accuracy, Agreement

ASHA – accredited social health activists, ANM – auxiliary nursing midwife, BPHN – block level nursing staff, ChARM – Children’s Automated Respiration Monitor, CHO – community health officer, CHW – community health worker, FCHV – female community health volunteer, FLHFW – front line health facility worker, HEW – health extension workers, HW – health worker, RR count – respiratory rate count, VEP – video expert panel

The studies were conducted across diverse settings, including hospitals (inpatient, outpatient, emergency, and triage units), primary health facilities, and community contexts. Study participants included children aged 0–59 months, community health workers (CHWs), health extension workers (HEWs), frontline health facility workers (FLHFWs), physicians, nurses, and caregivers.

The devices were operated by different cadres of health workers depending on the study design. In diagnostic accuracy studies, the reference standard most commonly consisted of manual RR counts performed by trained nurses, paediatricians, or expert physician panels, with assessments conducted either simultaneously or sequentially with device measurements.

Primary outcomes included diagnostic accuracy and agreement measures (such as sensitivity, specificity, and concordance with the reference standard), as well as implementation outcomes including usability, acceptability, and considerations for scale-up.

### Risk of bias

**Figure 2:**
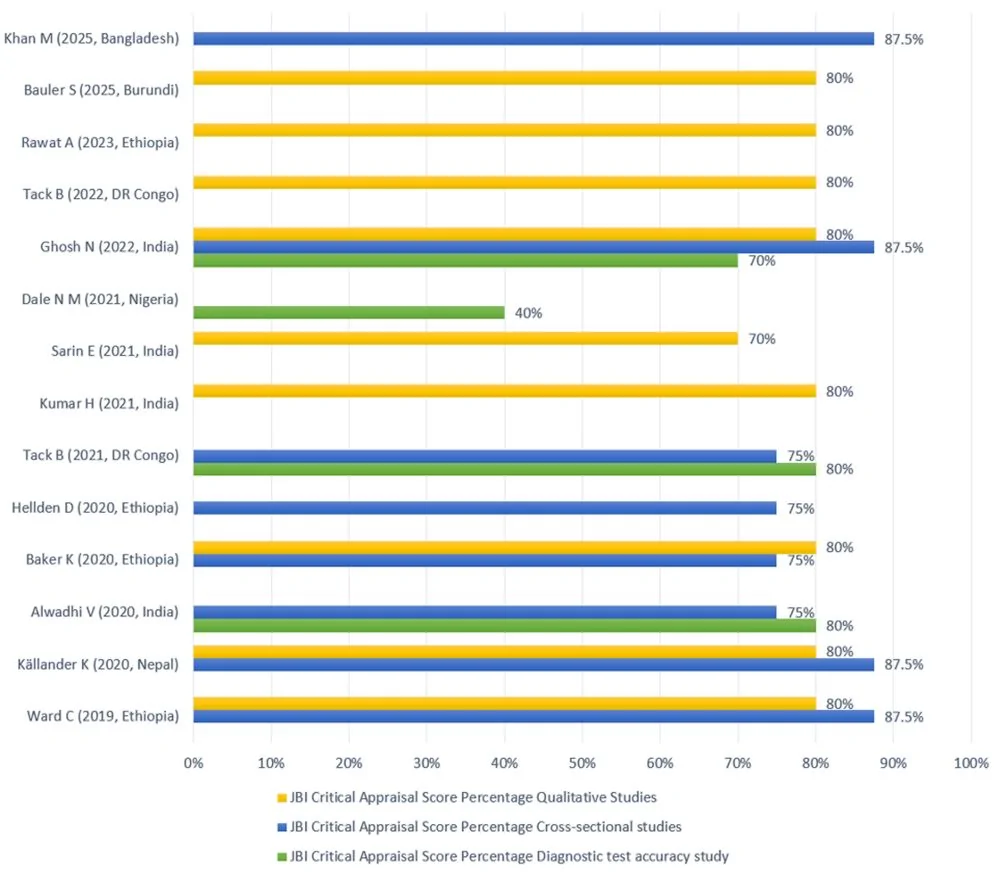
Risk of bias assessments using the Joanna Briggs Institute (JBI) tool.

According to the JBI critical appraisal checklists, most studies met a substantial proportion of the relevant appraisal criteria, although several recurring methodological limitations were identified across the evidence base. Diagnostic accuracy studies [30–33] generally showed acceptable quality, though limitations included a lack of blinding in RR assessment, incomplete reporting of participant selection procedures, and variation in reference standards used for comparison. Cross-sectional and mixed-methods studies generally met most appraisal criteria, although concerns regarding representativeness and reliance on convenience sampling were noted in several studies. One study quality had more substantial methodological limitations (JBI score 4/10) due to the absence of a true gold standard, lack of appropriate exclusions, and no clearly defined reference standard for accurate classification [34]. Cross-sectional and mixed-methods studies met most appraisal criteria, with occasional concerns related to representativeness of the sample and reliance on convenience sampling. Qualitative studies [20,35,36] demonstrated clear alignment between research questions, data collection, and analysis, though some lacked detail on researcher reflexivity or participant recruitment strategies. The distribution of JBI critical appraisal scores is illustrated in **Figure 2**, while the detailed item-level assessments for each study are presented in Table S4 in the **Online Supplementary Document****.**

### Description of the devices

#### Masimo Rad-G

**Figure 3:**
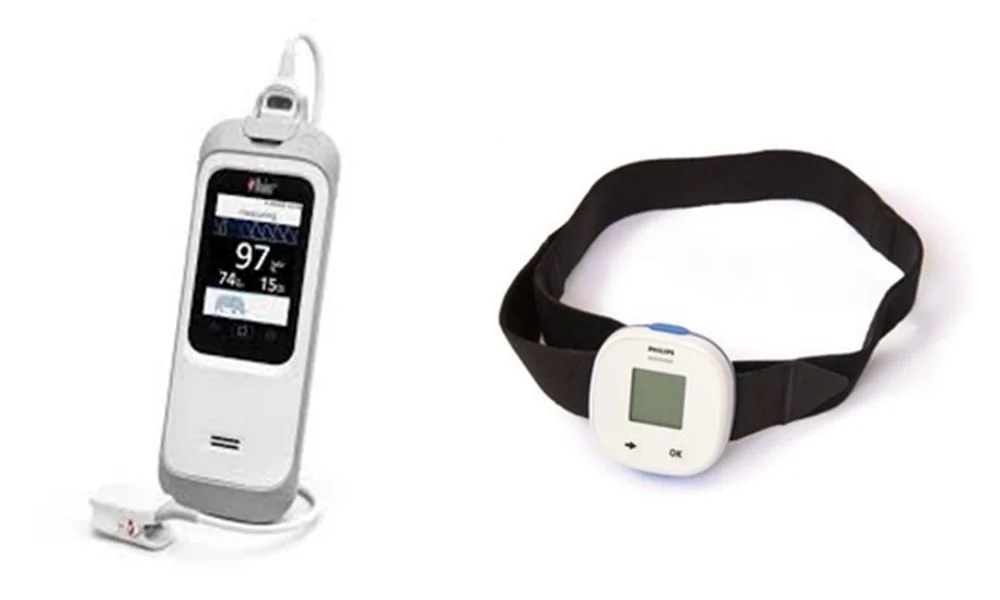
Masimo Rad-G device (left); Children’s Automated Respiration Monitor (ChARM) device (right).

Masimo has designed a handheld finger-tip multimodal device named the Rad-G (**Figure 3**). It is a low-cost, robust device with a rechargeable battery. This device is designed primarily for pneumonia screening with spot-checking SpO_2_ and RR and automated pneumonia treatment decision-making in low-resource settings. The development of the device is supported by the Bill & Melinda Gates Foundation (BMGF) [37]. It uses the photoplethysmogram (PPG), a method for estimating RR in real time, obtained from pulse oximetry [31].

#### ChARM

Philips has developed ChARM (**Figure 3**), which can convert chest movements detected by accelerometers into a precise breathing count using specially designed algorithms. The technology is based on the Intellivue cableless monitor, which comprises 3D accelerometry and signal processing of respiration-based biomechanics. The device is tied around the child’s belly, automatically counts RR and classifies fast breathing according to the WHO guidelines. It is specially designed for resource-constrained settings. This device is rechargeable, and its operation time depends on its use. The water and dust resistance feature makes it suitable for rural areas' environmental conditions and ensures long-term use. This device has CE-marking and is commercially available [38].

### Quantitative synthesis

#### Accuracy

Five studies reported quantitative data on the diagnostic performance or agreement of automated RR counters compared to manual or expert reference standards (**Table 2**). These were conducted mainly in hospital outpatient or inpatient settings, with sample sizes ranging from 97 to 514 children.

**Table 2:** Diagnostic performance of automated respiratory rate counters compared to reference standards

Study ID	Settings	Device	Sample Size	True Positive	False Positive	True Negative	False Negative	Sample Prevalence	Sensitivity (95% CI)	Specificity (95% CI)	Agreement (95% CI)	Kappa
Alwadhi V, 2020	Hospital-outpatient	Masimo Rad-G	97	62	2	30	3	67.0%	95.4% (90.3–100.0)	93.8% (85.4–100.0)	94.8% (90.4–99.2)	0.85
Tack B, 2021	Hospital-inpatient	Masimo Rad-G	202	66	2	113	21	43.1%	75.9% (66.9–84.9)	98.3% (95.9–100.0)	88.6% (84.2–93.0)	0.76
Dale NM, 2021	Hospital-inpatient	Masimo Rad-G	514	Not reported	Not reported	Not reported	Not reported	Not reported	Not reported	Not reported	84%	0.55–0.68
Ghosh N, 2022	Community setting	ChARM	132	Not reported	Not reported	Not reported	Not reported	Not reported	Not reported	Not reported	Not reported	0.74
Khan M, 2025	Hospital-outpatient	ChARM	257	68	12	174	3	24.1%	95.8% (91.1–100.0)	93.5% (90.0–97.1)	94.2% (91.3–97.0)	0.86

CI – confidence interval

The Masimo Rad-G demonstrated variable accuracy across studies but consistently high specificity and reasonable agreement. In Alwadhi *et al.*., sensitivity was 95.4% and specificity was 93.8%, with an overall agreement of 94.9% and a kappa of 0.85, indicating excellent concordance [31]. In Tack *et al*., sensitivity was lower at 75.9%, although specificity was very high (98.3%) [32]. Agreement reached 88.6% with a kappa of 0.76, reflecting substantial concordance. In Dale *et al*., the Masimo Rad-G showed moderate agreement with nurse-measured RR in malnourished inpatients, with kappa values of 0.68 among older (12–59 months) and 0.55 among younger (6–11 months) children, and an overall classification agreement of 84% [34].

The ChARM device also showed variable findings. In Khan *et al.*, the sensitivity was 95.8%, specificity 93.5%, agreement 94.2%, and kappa 0.86, indicating high concordance with the reference measurements [30]. In contrast, Ghosh *et al*. reported overall moderate agreement between automated and manual measurements, with a kappa value of 0.74 [39]. Both studies reported that diagnostic accuracy or agreement was higher in older children (12–59 months) compared with younger children (<12 months).

Interpretation of these performance estimates should be undertaken cautiously because comparator methods varied across studies and no universally accepted gold standard for RR measurement was used.

#### Usability

Four studies conducted in India, Nepal, and Ethiopia reported the usability of the ChARM device. Across these studies, the device demonstrated moderate-to-high usability, although performance depended strongly on user training and adherence to recommended procedures. In Ghosh *et al*., community health workers in India achieved correct child positioning in 52% of assessments, correct device positioning in 72.1%, child calmness in 78%, and appropriate classification in 71%, with most assessments completed on the first attempt [39]. Källander *et al*. in Nepal and Ward *et al*. in Ethiopia reported improvements in procedural adherence over time, with correct device placement and classification exceeding 90% in most observations, although full adherence to WHO and manufacturer steps remained variable [40,41]. Baker *et al*. similarly reported high usability, improved compliance after training, and reduced reading time, with motion artefacts identified as the main cause of failed measurements [42]. Overall, ChARM was feasible for use in community settings, but optimal performance depended on adequate training, procedural adherence, and minimising child movement during assessment.

#### Time efficiency

Khan et al. reported that the median time required to measure RR using the ChARM device was 66 seconds [30]. The time needed to complete measurements varied depending on child behaviour and RR, with longer measurement times observed when children were moving or crying, indicating increased time demand under less optimal measurement conditions.

### Qualitative synthesis

Thematic synthesis generated three interrelated descriptive themes relating to perceived accuracy, perceived usability, and perceived acceptability and impact. Given the implementation-focused nature of the included studies and the limited depth of conceptual reporting in some studies, the synthesis primarily identified descriptive patterns across studies while also providing insights relevant to the implementation of automated RR counters in LMIC settings. The three themes are presented in turn with illustrative quotes in Table S5 in the **Online Supplementary Document**, with the number of studies supporting each theme.

#### Perceived accuracy

Two studies assessing the ChARM device reported that health workers consistently valued its ability to deliver accurate readings, which, in turn, enhanced their confidence in clinical decision-making compared with manual RR counting [20,41]. In addition, ChARM was credited in two studies with improving the accuracy of case classification, helping to minimise diagnostic errors and reduce false assessments. Caregivers also responded positively to the light sensor feature, appreciating the intuitive, color-coded signals that allowed even those with limited literacy to understand the results, thereby fostering greater trust in the health assessment process [39,41].

In contrast, health workers highlighted that the Masimo Rad-G device delivered faster, more reliable results than manual methods. Providers described it as an efficient, one-step tool that simplified case management and improved clinical accuracy [20,36,42]. Masimo Rad-G improved the ability of health workers to classify pneumonia cases with greater precision, moving beyond guesswork associated with manual counting. The devices offered age-specific readings and clear referral signals, such as red lights, that guided decisions on whether a child required higher-level care. Job aids further supported ease of classification, making referral decisions more straightforward [20,42,43].

#### Perceived usability

Usability of the devices was influenced by both contextual challenges and design features, with several studies highlighting factors that affected their practical application in routine clinical settings. Device sustainability emerged as a concern. One study described problems with the ChARM device’s battery life and expressed mixed preferences regarding rechargeable *vs.* non-rechargeable models, citing concerns about electricity fluctuations in health centres and the need for a reliable power supply [20]. In two studies, health workers noted concerns about battery reliability and maintenance of the Masimo Rad-G device [20,36]. While another study found that most community health workers rarely needed to recharge their devices for over eight weeks, others reported problems linked to electricity shortages, device malfunctions, and the need to monitor charging, which at times made the devices unusable. Recommendations included providing portable backup battery packs and improving the design to ensure uninterrupted use in low-resource settings [20,36].

In two studies, health workers reported that child agitation and distress, particularly when attaching the device (ChARM) or during illness, often prolonged consultations and made measurements more difficult [20,41]. Similarly, four studies further reported that child movement, crying, or fear of the device (Masimo Rad-G) often led to errors or extended assessment times, although some health workers observed that distraction features such as games or images could help calm older children and facilitate readings [20,32,36,42].

User feedback across studies revealed a mix of positive and negative perceptions of device features, with specific design elements influencing both usability and confidence in device operation. In two studies, caregivers and health workers found the ChARM device’s red and green light indicators effective for communicating results clearly; however, some health workers initially expressed concern that the device might be too complex or difficult to operate [40,41]. In contrast, several studies identified specific usability limitations with the Masimo Rad-G device. These included initial apprehension about unfamiliar light displays, difficulty securing probes, and interface issues that led to accidental errors, particularly in age selection [36,42]. Health workers also reported uncertainty about interpretation thresholds and a lack of training in functions such as pulse rate measurement, which undermined their ability to use the device fully [20,36]. Nonetheless, some cited the Masimo Rad-G’s compact size, weight, and ease of cleaning as practical advantages; some staff reported that its alarm sounds increased child agitation and led caregivers to associate the device with severe illness and poor prognosis [32].

In two studies, healthcare providers noted that ChARM saved time by giving immediate readings, though some reported the need for complete attention during use limited multitasking [20,40]. In four studies, healthcare providers reported that Masimo Rad-G saved time by enabling quick, automatic detection of fast breathing and supporting rapid triage. However, some also noted that prolonged or repeated measurement times disrupted patient flow, highlighting variability in efficiency across settings [32,35,36,43].

#### Perceived acceptability and impact

Initial hesitation toward device use was common among both health workers and caregivers, but with training and repeated use, confidence increased and the devices became more acceptable and user-friendly across settings. In several studies, health workers described initial fear or discomfort in using the ChARM device, often finding it difficult or intimidating at first. With training and practice, however, most gained confidence, reporting that the devices became user-friendly and acceptable over time [39–41]. Similarly, across two studies, both caregivers and health workers reported initial hesitation or fear toward Masimo Rad-G with children often reacting with distress at first sight of the device. However, most children calmed once the probe was applied, and caregivers valued the technology for enabling quicker, more accurate diagnosis [20,36]. Health workers overcame early concerns through training and practice, gaining confidence and finding the devices easier to use over time [42].

The devices were reported to have a broader impact beyond clinical accuracy, influencing caregiver trust, provider motivation, and preferences for continued use in routine care. In two studies, frontline health workers noted that the ChARM device had a broader impact by strengthening caregivers’ trust in their services and motivating continued use of the devices. Health workers expressed a desire to use the devices in the future, citing the high burden of pneumonia and the number of affected children in their communities [40,41]. In three studies, both health workers and caregivers expressed clear preferences for the Masimo Rad-G device over manual counting. Providers valued the devices for reducing bias and simplifying measurements, while caregivers reacted positively, often requesting their use and associating them with quicker recovery and more reliable care [20,36,42].

#### Considerations for scale-up

Across studies, participants emphasised that successful scale-up required addressing training, supervision, and health system readiness. Devices were valued for their potential to standardise pneumonia diagnosis and strengthen referral systems, but sustained implementation would depend on cost, durability, and integration into existing community health platforms.

## DISCUSSION

This systematic review examined the performance and implementation of automated RR counters for the assessment of pneumonia in children under five in LMIC settings. The qualitative synthesis primarily generated descriptive themes relating to perceived accuracy, usability, acceptability, and implementation considerations, which were interpreted alongside the limited quantitative evidence. The synthesis also identified practical limitations that warrant consideration during implementation.

### Accuracy and confidence in decision-making

Accurate measurement of RR is critical for the early and reliable detection and classification of pneumonia, as minor counting errors can result in misclassification of tachypnea, thereby affecting accurate diagnosis and clinical decision-making [44]. Qualitative findings suggest that ChARM and Masimo Rad-G were perceived by the users to provide more reliable RR measurement compared to manual counting. ChARM was noted to improve confidence in case classification and was perceived to reduce diagnostic errors, while Masimo Rad-G was often perceived as faster and more dependable, offering age-specific classification support and referral prompts that assisted clinical decision making.

These perceptions were consistent with limited quantitative evidence identified in this review. In one study, Masimo Rad-G showed high sensitivity and specificity, with agreement rates exceeding 90% and kappa values indicating substantial agreement [31]. Similarly, one study reported over 94% agreement for ChARM compared with the reference standard [30]. The convergence between users’ perceptions of improved confidence, classification, and decision-making and the quantitative diagnostic performance findings provides preliminary support for the potential utility of these devices in routine care. However, the available evidence remains exploratory, and larger independent studies conducted under routine implementation conditions are needed to determine whether the perceived and measured benefits are sustained across diverse LMIC settings.

### Usability challenges

Several challenges emerged that could affect the sustained use and effectiveness of automated RR counters in low-resource settings. Key barriers included unreliable battery performance and inconsistent access to electricity, which at times rendered devices unusable or required close monitoring. Design-related concerns were particularly noted with Masimo Rad-G, whereas some users initially found ChARM device complex to operate. Challenges such as the need for focused attention during ChARM use and prolonged or repeated measurements with Masimo Rad-G due to child movement or device errors were reported to disrupt clinical workflow and patient flow. Similar battery and time-related challenges were reported in a previous evaluation of the semi-automated mPneumonia device in low-resource settings [45], highlighting that operational constraints are not device-specific, but may reflect broader contextual realities.

A lack of training on specific functions further reduced user confidence and restricted full utilisation of available features. While both ChARM and Masimo Rad-G were generally perceived as time-saving tools, their efficiency was highly context-dependent. Overall, the available evidence suggests that real-world usability may be influenced by device robustness, interface design, and the availability of user support mechanisms such as training and job aids. Even technically high-performing devices may underperform in routine practice if the users perceive them as cumbersome, fragile, or confusing. Moreover, healthcare providers in routine settings have been reported to deviate from WHO guidelines, resulting in incomplete and imprecise clinical assessments [46,47]. Embedding structured training programmes, such as simulation-based modules, regular refresher sessions, on-site mentorship, and competency evaluations may help reinforce adherence to standardised protocols.

Child agitation during measurement was another widespread issue, often resulting in prolonged consultations or inaccurate readings. An additional consideration is the potential influence of age on usability and performance. Some included studies reported better agreement in older children than in younger infants, although age-specific findings were not consistently reported across studies. This observation is plausible given the greater likelihood of movement during assessment, smaller tidal volumes, and increased respiratory variability in younger children, all of which may make obtaining stable respiratory rates more challenging. Importantly, infants represent one of the groups at highest risk of severe pneumonia and pneumonia-related mortality. Consequently, difficulties in obtaining accurate measurements in this age group may have important implications for clinical use. Future studies should evaluate age-specific performance and usability more systematically to determine whether additional training, device adaptations, or implementation strategies are needed for younger children. Addressing these barriers through targeted design improvements, enhanced user support and training, and infrastructure planning may facilitate implementation and help maximise device performance in routine practice.

### Time efficiency issues

Only one study assessed the time required for RR measurement using an automated device [30]. This study reported a median measurement time of 66 seconds with the ChARM device, suggesting a reasonable duration for routine clinical use. However, measurement time varied with child behaviour, with longer durations observed when children were moving or crying. Importantly, the reported time excluded device attachment and preparation, which may increase total workflow time in real-world settings. These findings suggest that the efficiency of automated devices is likely influenced by both technical performance and contextual factors, including child cooperation, user experience, and workflow integration. These additional, often unmeasured, time demands should be considered during implementation planning, as they can affect patient flow, staff workload, and overall efficiency in low-resource settings.

### Impact and implementation

In 2014, a panel of global experts ranked the identification and evaluation of novel diagnostic tools to enhance pneumonia classification at the community level as the fifth overall research priority and the second in terms of importance and potential impact [48]. This review systematically identified and synthesised the currently available evidence on automated RR counters for identifying childhood pneumonia in LMIC settings. Although the number of eligible studies was limited and the evidence base was heterogeneous, this itself is an important finding, highlighting the early stage of evidence generation and the substantial research gap in this field. In emerging areas of diagnostic innovation, systematic reviews remain valuable for collating fragmented evidence, identifying consistent signals, and informing priorities for future investigation. Accordingly, the findings of this review should be interpreted as mapping the current evidence base and identifying implementation considerations rather than providing definitive guidance for large-scale adoption.

The qualitative evidence suggested that both ChARM and Masimo Rad-G were generally viewed positively by healthcare providers and caregivers. ChARM was reported to enhance caregiver trust in health services and increase provider motivation to use improved diagnostic tools, while Masimo Rad-G was often preferred over manual counting because of its perceived simplicity, reduced observer bias, and support for clinical decision-making. These perceptions were broadly consistent with the available quantitative findings, although evidence remains limited and derived from a small number of studies.

Collectively, these available findings indicate potential of automated RR counters to provide a more accurate and consistent respiratory rate that supports timely pneumonia assessment in low-resource settings, although the evidence remains preliminary and further validation is required.

Accordingly, automated RR counters should currently be viewed as promising adjunctive tools rather than established scale-up solutions. Further high-quality, independently conducted studies across diverse routine settings are needed to confirm effectiveness, sustainability, cost-effectiveness, and optimal implementation strategies. In parallel, successful broader implementation will require attention to key operational considerations, including adequate training and supervision of healthcare personnel, reliable maintenance and supply systems, and effective integration into routine clinical and community workflows.

### Strengths and limitations

A key strength of this review is the systematic synthesis of both quantitative and qualitative evidence on automated RR counters for the assessment of RR to support identifying childhood pneumonia in LMIC settings. By integrating diagnostic performance findings with perspectives from healthcare workers across multiple LMIC contexts, the review provides a detailed understanding of both technical accuracy and real-world operational considerations. Additional strengths include prospective protocol registration, adherence to PRISMA guidance, and formal risk-of-bias appraisal across multiple study designs.

Several limitations should be considered. First, the available evidence base remains relatively limited, with a modest number of eligible studies and only five reporting quantitative diagnostic performance outcomes with substantial heterogeneity existing across study designs, populations, comparator standards, outcome definitions, and reporting methods, which precluded meta-analysis. Nevertheless, narrative synthesis enabled identification of consistent patterns and context-specific findings. Second, the published literature primarily evaluated two available automated devices (Masimo Rad-G and ChARM), so applicability to other emerging technologies that are not automated remains limited. Third, the review was restricted to English-language publications. Although the systematic search did not identify any eligible non-English studies and included studies from multiple countries across Africa and Asia, it remains possible that relevant non-English or grey literature was not captured, particularly locally generated implementation evidence from LMIC settings. Fourth, qualitative studies varied in reporting depth, limiting higher-order interpretive synthesis, however, sufficient data were available to generate informative descriptive themes relating to usability, acceptability, and implementation barriers, *etc*. Overall, the evidence base is emerging, but this review provides a timely synthesis of current knowledge and highlights priorities for future research. In addition, one included study reported support from a device manufacturer, and some evaluations were conducted within broader implementation initiatives supporting the assessment of novel RR technologies. Although this does not necessarily compromise study validity, the potential influence of sponsorship, developer involvement, or selective reporting cannot be entirely excluded and should be considered when interpreting the findings.

### Recommendations

Future mixed-methods studies should explicitly report quantitative and qualitative findings separately while maintaining clear linkage between evidence streams to facilitate transparent synthesis and interpretation. Studies should also examine how performance varies by age group, illness severity, child movement, staffing levels, and patient volume. Future studies should also adopt standardised reference standards and harmonised reporting outcomes to improve comparability and strengthen confidence in diagnostic performance estimates across settings. Devices should be piloted in real-world clinical workflows to assess effects on patient flow, staff multitasking demands, and contingency for measurement failures. To improve comprehensiveness and better capture locally generated evidence from LMIC settings, multilingual search strategies and targeted grey-literature sources, particularly in French, Hindi, Amharic, Nepali, and other relevant languages, should be incorporated. For device developers, priorities include battery and charging resilience, probe stability, intuitive interfaces, clear decision-support displays, reduced sensitivity to child movement, and child-friendly design features that minimise distress during measurement.

## CONCLUSIONS

Automated RR counters show promising early evidence for supporting RR assessment to support timely pneumonia assessment and diagnosis in LMIC settings. However, the current evidence base remains limited and heterogeneous. Further high-quality studies across diverse routine-care settings are needed to evaluate effectiveness, sustainability, cost-effectiveness, and implementation feasibility before broad scale-up recommendations can be made.

## Additional material


Online Supplementary Document

## Data Availability

**Data availability:** All data used in this study are contained within the article and its **Online Supplementary Document**.
