# Supplementary material for: Performance of automated respiratory rate counters for respiratory rate assessment to support pneumonia detection among children under five years of age in low- and middle-income settings: a systematic review of qualitative and quantitative evidence
Source: J Glob Health. 2026 Aug 21;16:04281. doi: 10.7189/jogh.16.04281 (PMC13495544; doi:10.7189/jogh.16.04281)
Supplement: Supplementary file 1 [file jogh-16-04281-s001.pdf]

Ibrahim SB, Haque KS, Ahmed MZEMNU, Uddin MJ, Ahmed S, Sultana S, Khan AM. Performance of automated respiratory rate counters for respiratory rate assessment to support pneumonia detection among children under five years of age in low- and middle-income settings: a systematic review of qualitative and quantitative evidence. J Glob Health. 2026;16:04281.

Table S1. Search strategies

**A. Name of Database: Ovid MEDLINE(R) (2014-01-01 to 2025-07-15)**		
1	exp Pneumonia/	402605
2	pneumoni*.mp.	333139
3	exp Respiratory Tract Infections/	695270
4	Tachypnea/	334
5	(tachypn* or "respiratory tract infection*" or "acute respiratory infection*" or ari or "acute respiratory tract infection*" or arti or "lower respiratory tract infection*" or lrti).mp.	77849
6	breath* count*.mp.	121
7	fast breath*.mp.	377
8	"febrile illness".mp.	6287
9	1 or 2 or 3 or 4 or 5 or 6 or 7 or 8	892964
10	exp Child/	2277492
11	exp Infant/	1314423
12	exp Infant, Newborn/	706949
13	exp Child, Preschool/	1040565
14	(child* or preschool or newborn or infant* or neonat* or pediatric or paediatric).mp.	3882029
15	(("under five" or "under 5") adj child*).mp.	2742
16	10 or 11 or 12 or 13 or 14 or 15	3882029
17	Respiratory Rate/	4374
18	Respiration/	83155
19	("respiratory rate monitor*" or "respiratory monitoring" or "respiratory rate" or "respiratory monitor*" or "respiratory rate count*" or "respiratory count*" or "automated respiratory rate" or "RR count*" or "automated respiratory count*" or "automatic respiratory monitor" or "ARI diagnostic device" or "automatic respiratory monitor" or "automated respiratory rate count*" or breath* count* or breath* rate).mp.	23376
20	17 or 18 or 19	102109
21	9 and 16 and 20	1233
22	limit 21 to yr="2014 -Current"	475
**B. Name of Database: Embase 2014-01-01 to 2025-07-15**		
1	exp Pneumonia/	480843
2	pneumoni*.mp.	631674
3	exp Respiratory Tract Infections/	660661
4	Tachypnea/	27321
5	(tachypn* or "respiratory tract infection*" or "acute respiratory infection*" or ari or "acute respiratory tract infection*" or arti or "lower respiratory tract infection*" or lrti).mp.	202416
6	breath* count*.mp.	197
7	fast breath*.mp.	548
8	"febrile illness".mp.	9646
9	1 or 2 or 3 or 4 or 5 or 6 or 7 or 8	1166348
10	exp Child/	3907660
11	exp Infant/	1461930

12	exp Infant, Newborn/	758110
13	exp Child, Preschool/	752277
14	(child* or preschool or newborn or infant* or neonat* or pediatric or paediatric).mp.	5101401
15	(("under five" or "under 5") adj child*).mp.	3315
16	10 or 11 or 12 or 13 or 14 or 15	5138963
17	Respiratory Rate/	82170
18	Respiration/	127166
19	("respiratory rate monitor*" or "respiratory monitoring" or "respiratory rate" or "respiratory monitor*" or "respiratory rate count*" or "respiratory count*" or "automated respiratory rate" or "RR count*" or "automated respiratory count*" or "automatic respiratory monitor" or "ARI diagnostic device" or "automatic respiratory monitor" or "automated respiratory rate count*" or breath* count* or breath* rate).mp.	92628
20	17 or 18 or 19	211264
21	9 and 16 and 20	5080
22	limit 21 to yr="2014 -Current"	3546
**C. Name of database: Web of Science Core Collection (2014-01-01 to 2025-07-15)**		
1	TS=(pneumoni* or tachypn* or "respiratory tract infection*" or "acute respiratory infection*" or ari or "acute respiratory tract infection*" or arti or "lower respiratory tract infection*" or lrti)	395640
2	TS=("breath* count*")	120
3	TS=("fast breath*")	376
4	TS=("febrile illness")	6253
5	#1 OR #2 OR #3 OR #4	401674
6	TS=(child* or preschool or newborn or infant* or neonat* or pediatric or paediatric)	3881792
7	TS=(("under five" or "under 5") NEAR child*)	12863
8	#6 OR #7	3881792
9	TS=("respiratory rate monitor*" or "respiratory monitoring" or "respiratory rate" or "respiratory monitor*" or "respiratory rate count*" or "respiratory count*" or "automated respiratory rate" or "RR count*" or "automated respiratory count*" or "automatic respiratory monitor" or "ARI diagnostic device" or "automatic respiratory monitor" or "automated respiratory rate count*" or breath* count* or breath* rate.)	63133
10	#5 AND #8 AND #9	1199
11	#5 AND #8 AND #9 Timespan: 2014-01-01 to 2025-07-15	693
**D. Name of database: Scopus (2014-01-01 to 2025-07-15)**		
1	((TITLE-ABS-KEY (pneumoni* or tachypn* or "respiratory tract infection*" or "acute respiratory infection*" or ari or "acute respiratory tract infection*" or arti or "lower respiratory tract infection*" or lrti) OR TITLE-ABS-KEY ("breath* count*") OR TITLE-ABS-KEY ("fast breath*") OR TITLE-ABS-KEY ("febrile illness"))) AND ((TITLE-ABS-KEY (child* or preschool or newborn or infant* or neonat* or pediatric or paediatric)) OR (TITLE-ABS-KEY (("under five" or "under 5") W/0 child*))) AND (TITLE-ABS-KEY ("respiratory rate monitor*" or "respiratory monitoring" or "respiratory rate" or "respiratory monitor*" or "respiratory rate count*" or "respiratory count*" or "automated respiratory rate" or "RR count*" or "automated respiratory count*" or "automatic respiratory monitor" or "ARI diagnostic device" or "automatic respiratory monitor" or "automated respiratory rate count*" or breath* count* or breath* rate)) AND PUBYEAR > 2013	2532

Table S2. Extraction of data from eligible studies

**A. Identification of the study**		
1	Study ID	
2	First author	
3	Year of publication	
4	Title	
5	Name of the journal	
6	Study Region – Country	
7	Outcome Measure	
**B. Study eligibility**		
8	Eligibility- Yes/ No	
9	Reason for exclusion	
10	Comment	
**C. Study methods**		
11	Study Settings	
12	Study Design	
13	Study Method	
14	Study period	
15	Sampling method	
**D. Population characteristics**		
16	Population	
17	Sample size	
18	Number of observations	
19	Inclusion criteria	
20	Exclusion criteria	
**E. Index test**		
21	Device test	
22	RR assessment done by	
23	Number of health workers	
24	Type of health workers	
25	Education	
26	Training	
27	Blinding	
**F. Reference standard**		
28	Reference standard used	
29	Qualification	
30	Blinding	
**G. Respiratory rate assessment**		
31	Sequence of the assessment	
32	Time interval between the assessments	
**H. JBI critical appraisal checklist (Diagnostic test accuracy study)**		
33	Was a consecutive or random sample of patients enrolled?	
34	Was a case-control design avoided?	
35	Did the study avoid inappropriate exclusions?	
36	Were the index test results interpreted without knowledge of the results of the reference standard?	
37	If a threshold was used, was it pre-specified?	

38	Is the reference standard likely to correctly classify the target condition?	
39	Was there an appropriate interval between index test and reference standard?	
40	Did all patients receive the same reference standard?	
41	Were all patients included in the analysis?	
42	applicability concern	
43	Critical appraisal score	
**I. JBI critical appraisal checklist (Cross-sectional studies)**		
44	Were the criteria for inclusion in the sample clearly defined?	
45	Were the study subjects and the setting described in detail?	
46	Was the exposure measured in a valid and reliable way?	
47	Were objective, standard criteria used for measurement of the condition?	
48	Were confounding factors identified?	
49	Were strategies to deal with confounding factors stated?	
50	Were the outcomes measured in a valid and reliable way?	
51	Was appropriate statistical analysis used?	
52	Critical appraisal score	
**J. JBI critical appraisal checklist (Qualitative studies)**		
53	Is there congruity between the stated philosophical perspective and the research methodology?	
54	Is there congruity between the research methodology and the research question or objectives?	
55	Is there congruity between the research methodology and the methods used to collect data?	
56	Is there congruity between the research methodology and the representation and analysis of data?	
57	Is there congruity between the research methodology and the interpretation of results?	
58	Is there a statement locating the researcher culturally or theoretically?	
59	Is the influence of the researcher on the research, and vice versa, addressed?	
60	Are participants, and their voices, adequately represented?	
61	Is the research ethical according to current criteria, or for recent studies, and is there evidence of ethical approval by an appropriate body?	

62	Do the conclusions drawn in the research report flow from the analysis or interpretation of the data?	
63	Critical appraisal score	
**K. Result (Accuracy of fast breathing identification)**		
64	Prevalence of fast breathing in the sample	
65	kappa (SE or 95% CI)	
66	True positive	
67	False positive	
68	True negative	
69	False negative	
70	Sensitivity (95% CI)	
71	Specificity (95% CI)	
72	Positive predictive value (95% CI)	
73	Negative predictive value (95% CI)	
74	Percent of accuracy or agreement (95% CI)	
75	Comment	
**L. Usability**		
76	Was the WHO guideline/IFU reinforced?	
77	Correct child positioning	
78	Device positioning	
79	Selection of the child's age on the device	
80	Motion of child (during) (calmness)	
81	Appropriate classification	

Figure S1. Steps of thematic synthesis

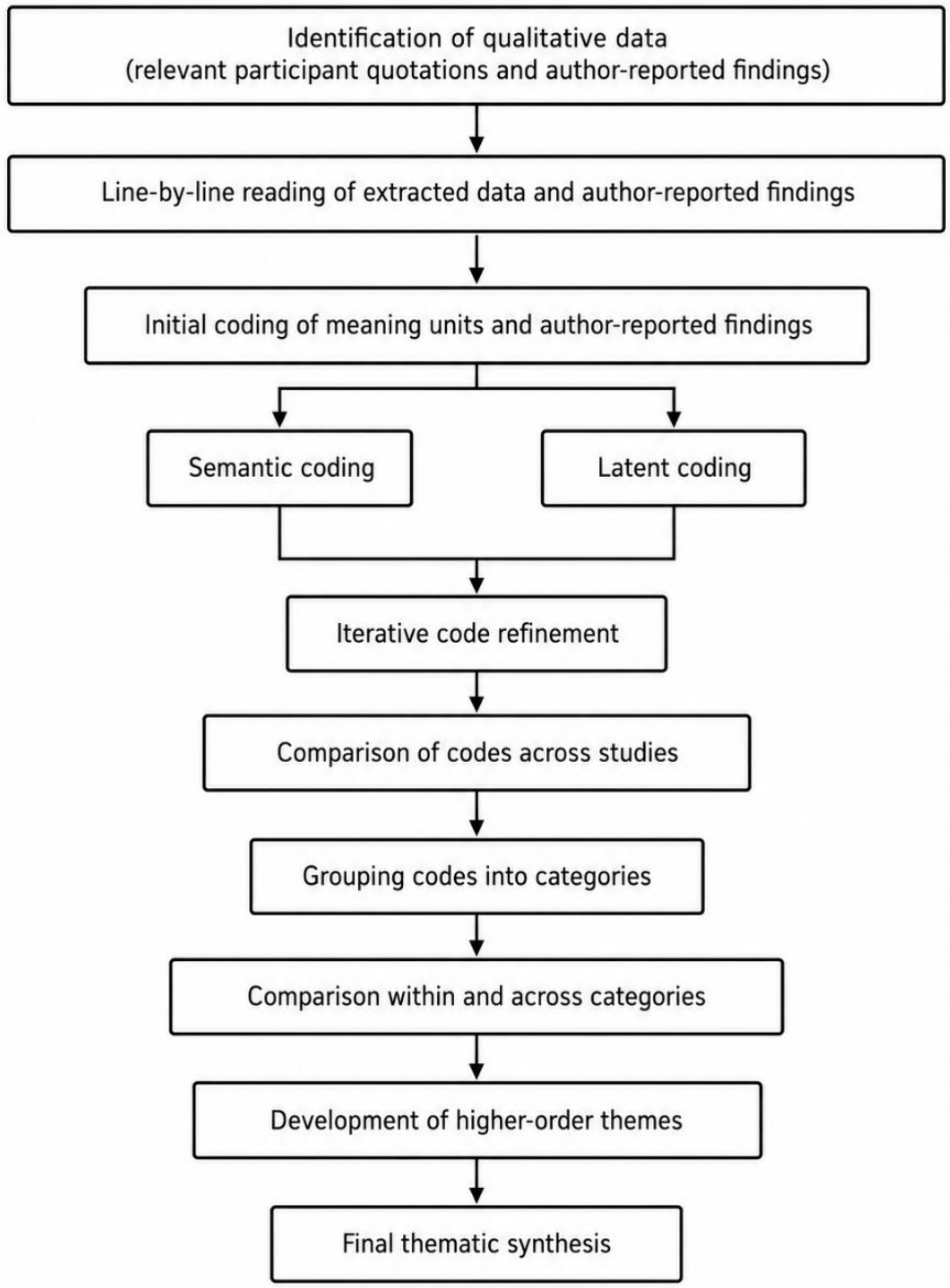

Table S3. Excluded studies with reasons for exclusion

#SL	Author	Year	Title	Reason for exclusion
1	Baker K	2015	Performance, Acceptability, and Usability of Respiratory Rate Timers and Pulse Oximeters When Used by Frontline Health Workers to Detect Symptoms of Pneumonia in Sub-Saharan Africa and Southeast Asia: Protocol for a Two-Phase, Multisite, Mixed-Methods Trial	Protocol only – describes planned two-phase multisite evaluation of respiratory rate timers and pulse oximeters, no results
2	Ginsberg A	2015	mPneumonia: Development of an Innovative mHealth Application for Diagnosing and Treating Childhood Pneumonia and Other Childhood Illnesses in Low-Resource Settings	Ineligible device type– development and usability testing of mPneumonia app, no evaluation of automated respiratory counters
3	Baker K	2015	Acceptability of selected respiratory rate counters and pulse oximeters for use by frontline health workers in the detection of pneumonia symptoms in children in sub-Saharan Africa and Southeast Asia	Protocol only – poster describing planned field evaluation of RR counters and pulse oximeters, no results
4	ClinicalTrials.gov registry	2015	Camera-based Measurement of Respiratory Rates	Ineligible device type– evaluated camera/vision-based RR measurement system, not automated counters for childhood pneumonia
5	Baker K	2015	Accuracy of selected devices for the detection of pneumonia symptoms-results from laboratory testing and field evaluations of respiratory rate counters and pulse oximeters with frontline health workers in sub-Saharan Africa and Southeast Asia	Ineligible device type– evaluated RR timers and pulse oximeters, not automated respiratory counters
6	Ginsburg A	2016	mPneumonia, an Innovation for Diagnosing and Treating Childhood Pneumonia in LowResource Settings: A Feasibility, Usability and Acceptability Study in Ghana	Ineligible device type– feasibility/usability pilot of mPneumonia app, no evaluation of automated respiratory counters
7	Sola J	2016	Towards an unsupervised device for the diagnosis of childhood pneumonia in low resource settings: automatic segmentation of respiratory sounds	Prototype/innovation – describes development of unsupervised respiratory sound segmentation tool, not evaluation of automated respiratory counters
8	Sakti N A	2016	Infant breathing rate counter based on variable resistor for pneumonia	Prototype/innovation – engineering design of variable resistor–based infant RR counter, no clinical diagnostic evaluation
9	Muro F	2017	Variability of the respiratory rate measurements in children suspected with non-severe pneumonia Northeast Tanzania	Ineligible device type– study assessed inter-observer variability of manual/video RR counts, no automated counter evaluated
10	Spence H	2017	Childhood pneumonia diagnostics: community health workers' and national stakeholders' differing perspectives of new and existing aids	Ineligible device type– qualitative study on CHW and stakeholder perspectives of diagnostic aids, no evaluation of automated counters

#SL	Author	Year	Title	Reason for exclusion
11	ClinicalTrials.gov registry	2017	Acute Respiratory Infection Diagnostic Aids (ARIDA) for Children Under Five Years When Used in a Controlled Setting in Ethiopia: Study Protocol for Controlled Accuracy Evaluation	Protocol only – describes planned accuracy evaluation of ARIDA devices in Ethiopia, no results
12	Ward C	2017	Selecting a reference standard for evaluating respiratory rate devices to diagnose symptoms of pneumonia in children under 5: Lessons learned from resource-poor settings in sub-saharan africa and asia	Ineligible device type– methodological study on reference standards for RR evaluation, not assessment of automated counters
13	Shah r	2018	An integrated diagnostic device for neonatal sepsis and childhood pneumonia	Prototype/innovation report – describes development of integrated ChARM device for pneumonia/sepsis, no results
14	Turnbull H	2018	Development of a novel device for objective respiratory rate measurement in low-resource settings	Prototype/innovation – describes development and pilot testing of Respimometer device, not evaluation of automated respiratorycounters
15	Baker K	2019	Performance of Four Respiratory Rate Counters to Support Community Health Workers to Detect the Symptoms of Pneumonia in Children in Low Resource Settings: A Prospective, Multicentre, Hospital-Based, Single-Blinded, Comparative Trial.	Ineligible device type– evaluated manual/semi-manual RR timers and phone apps, not automated respiratory counters
16	Jagadev P	2019	Non-contact monitoring of human respiration using infrared thermography and machine learning	Ineligible device type– non-contact infrared thermography and machine learning prototype tested in adults, not automated respiratory counters to support detecting childhood pneumonia
17	Ward C	2019	What is a breath? Working towards an improved reference standard for counting respiratory rate to validate new automated pneumonia diagnostic aids for children under five	Ineligible device type– evaluated video annotation tool as a reference standard, not automated respiratory counters
18	Baker K	2020	Automated Respiratory Rate Counter to Assess Children for Symptoms of Pneumonia: Protocol for Cross-Sectional Usability and Acceptability Studies in Ethiopia and Nepal	Protocol only – describes planned usability/acceptability study of automated counter, no results
19	Ward C	2020	Determining the Agreement Between an Automated Respiratory Rate Counter and a Reference Standard for Detecting Symptoms of Pneumonia in Children: Protocol for a Cross-Sectional Study in Ethiopia	Protocol only – describes planned diagnostic accuracy study of automated counter, no results
20	Baker K	2020	Children's Automated Respiration Monitor (ChARM) for Child Pneumonia Diagnosis by Community Health Workers in Mali: Innovating ChARM's Role in Supervision, Training and Diagnosis, a Cluster Randomized Control Trial	Protocol only – cluster RCT of ChARM for CHW supervision and training in Mali

#SL	Author	Year	Title	Reason for exclusion
21	Ahmed S	2021	A study protocol for community use of digital auscultation to improve diagnosis of paediatric pneumonia in Bangladesh	Protocol only – describes planned community-based study of digital auscultation for pneumonia diagnosis, no results
22	Stanberry L	2021	Development of a mobile health tool to objectively measure respiratory distress in infants	Prototype/innovation – development study of mobile health VPG tool for infant respiratory monitoring, not diagnostic accuracy for pneumonia
23	Startil A	2021	Evaluating the Interrater Agreement and Acceptability of a New Reference Tool for Assessing Respiratory Rate in Children under Five with Cough and/or Difficulty Breathing.	Ineligible device type– evaluated a video-based reference tool for RR assessment, not an automated respiratory counter
24	Ellington L	2021	Health workers' perspectives of a mobile health tool to improve diagnosis and management of paediatric acute respiratory illnesses in Uganda: a qualitative study	Ineligible device type– qualitative study on mHealth decision-support tool (ALRITE), no evaluation of automated respiratory counters
25	Stanberry L	2021	Development of a mobile health tool to objectively measure respiratory distress in infants	Prototype/innovation – development of mobile health video plethysmography tool for infant respiratory monitoring, not automated counters for pneumonia
26	Khan A	2022	Developing a video expert panel as a reference standard to evaluate respiratory rate counting in paediatric pneumonia diagnosis: protocol for a cross-sectional study	Protocol only – describes planned development of video expert panel reference standard for respiratory rate evaluation, no results
27	Spurr R	2022	Performance and usability of a new mobile application for measuring respiratory rate in young children with acute lower respiratory infections	Ineligible device type– evaluated semiautomated mobile phone apps (tap-based counters), not automated respiratory rate counters to support pneumonia diagnosis
28	Khan SR	2023	Multi-Modal Portable Respiratory Rate Monitoring Device for Childhood Pneumonia Detection	Prototype/innovation – describes development and preliminary volunteer testing of textile glove RR monitor, no diagnostic accuracy evaluation in sick children
29	Rishvanth S K	2024	Device to Measure Respiratory Rate in Children, in Resource Poor Settings	Prototype/innovation – describes development and lab testing of ToF sensor RR device, not evaluation of automated counters in sick children

Table S4. Risk of bias assessments using the JBI tool

**Author (Year, Country)**	**Study Design**	**JBI Critical Appraisal Score**			**JBI Critical Appraisal Score Percentage**		
		**Diagnostic test accuracy study**	**Cross-sectional studies**	**Qualitative Studies**	**Diagnostic test accuracy study**	**Cross-sectional studies**	**Qualitative Studies**
Ward C (2019, Ethiopia)	Mixed method		7/8	8/10		87.5%	80%
Källander K (2020, Nepal)	Mixed method		7/8	8/10		87.5%	80%
Alwadhi V (2020, India)	Cross-sectional study	8/10	6/8		80%	75%	
Baker K (2020, Ethiopia)	Mixed method		6/8	8/10		75%	80%
Hellden D (2020, Ethiopia)	Descriptive study		6/8			75%	
Tack B (2021, DR Congo)	Cross-sectional study	8/10	6/8		80%	75%	
Kumar H (2021, India)	Qualitative			8/10			80%
Sarin E (2021, India)	Qualitative			7/10			70%
Dale N M (2021, Nigeria)	Prospective observational agreement study	4/10			40%		
Ghosh N (2022, India)	Mixed method	7/10	7 / 8	8 / 10	70%	87.5%	80%
Tack B (2022, DR Congo)	Qualitative			8/10			80%
Rawat A (2023, Ethiopia)	Qualitative			8/10			80%
Bauler S (2025, Burundi)	Qualitative			8/10			80%
Khan M (2025, Bangladesh)	Cross-sectional study		7/8			87.5%	

Table S5. Themes and example quotes

**Main (Overarching) theme**	**Categories**	**Device**	**Example quotes**
**Theme 1:** Perceived accuracy of automated devices	Accurate diagnosis	ChARM	1. *"The previous method could lead us to make a wrong decision. ChARM displays the correct count, is more accurate because it is supported with a diagnostic tool. It displays the correct figure/count - (*Rawat A. et al., 2023)<br>2. *"Many HEWs expressed relief in the perceived accurate diagnosis provided by the device"* - (Ward C et al., 2019)
		Masimo Rad-G	*1.* *"Other positive attributes noted by the study participants were perceived improvement in iCCM, accuracy of the device, variety of parameters, and the ability for the device to display results quickly." - (*Baueler S. et al., 2025)<br>*2.* *"Caregivers also perceived the device as a new technology that could help frontline health workers identify diseases more accurately and quickly"* - (Bauler S. et al., 2025)<br>3. *"The majority of health workers felt that Rad-G was more accurate and faster than visually counting RR, with some describing it as a one-step device" - (*Rawat A. et al., 2023)<br>4. *"They replied to me that, formerly their children were clinically assessed by unconfirmed diagnosis, but after the device was introduced, that they are happy when they see the respiration is being counted correctly for their children"- (Rawat A. et al., 2023)*<br>5. *"Caregivers indicated they would recommend the device to others, and the main reason was because the device 'identifies disease'… or helps 'rule out whether the child is positive or negative."* - (Baker K. et al., 2020)
	**Accurate classification and referral**	Charm	1. *"Recording was accurate and the light sensors helped in the classification of the cases"* - (Ghosh N. et al., 2022)<br>*2.* *"As CHWs are over-burdened with multiple tasks, the device seems promising in saving their workload without affecting quality of the recording or classification, potentially leading to referrals that are more authentic"* - (Ghosh N. et al., 2022)<br>*3.* *"especially the light sensor method… Even caregivers who were illiterate could see the signals and understand the clarity of management as per*

Main (Overarching) theme	Categories	Device	Example quotes
			the color-coded classification" - (Ghosh N. et al., 2022)<br>4. *"it helped 'minimise our mistakes', avoids 'false diagnosis' and 'correctly identifies and classifies' disease" - (Ward C. et al., 2019)*
		Masimo Rad-G	1. *"But the new device shows the readings according to age groups, which makes it preferable to use" - (*Rawat A. et al., 2023)<br>2. *"The device gives a signal/message/if the case needs referral – a red light. The device itself tells you to refer the case and also shows whether cases are manageable at health centre level" - (*Rawat A. et al., 2023)<br>3. *"Earlier we were mentioning cough as diagnosis but now we are maintaining register as pneumonia and no pneumonia"* - (Kumar H. et al.,2021)<br>4. *"Many HEWs and FLHFWs mentioned using the job aid to help with classification and referral, specifically mentioning how easy or straightforward it was to identify classification categories using the charts in the job aid"* - (Baker K. et al., 2020)<br>5. *"they reported that their ability to classify pneumonia cases had improved, and this was important to avoid 'guessing based on respiratory count' and the 'difficulties' associated with classifying based on counting breaths"* - (Baker K. et al., 2020))
**Theme 2:** Perceived Usability	**Time efficiency**	ChARM	1. *"Before ChARM, we used to count children's breathing using our eyes. But now ChARM made breath counting easier. It is faster than the previous method because it can count the child's breaths immediately" - (Rawat A. et al.,* 2023*)*<br>2. *"Most FCHVs said they were unable to carry out other work when using the ChARM device" - (*Källander K. et al., 2020)

Main (Overarching) theme	Categories	Device	Example quotes
		Masimo Rad-G	1. *"The most appealing attribute of the device was the ability to easily and quickly triage or diagnose a child, especially, a child with a fever, as many CHWs lack access to functioning thermometers and stopwatches (Chronos) to support the measurement of respiratory rate" - (*Bauler S. et al., 2025)<br><br>2. *"This helps in identifying breathing difficulty automatically as fast breathing can easily be detected through this device. It helps in instant and easy diagnosis"* - (Kumar H. et al.,2021)<br>3. *"The pulse oximeter is very useful. It doesn't take time like we used to take when counting respiratory rate, it is easy to use"*- (Sarine E. et al., 2021)<br>4. *"All end-users, including the one who used it daily, reported that they struggled with the multimodal oximeter, particularly because of the occasionally long measurement times for the respiratory rate which disrupted patient flow at the triage"* - (Tack B. et al. 2022)<br>5. *"it takes time then to take a reading as I have to take the reading a number of times"* - (Sarine E. et al., 2021)
	**Battery Life Concerns**	ChARM	*1. "Some felt the rechargeable devices allowed for continued use; others felt the non-rechargeable devices were better, as they were not influenced by the electricity fluctuations in the health centre" - (Rawat A. et al.,* 2023*)*
		Masimo Rad-G	1. *"Most CHWs did not need to recharge their oximeters over the 8-week pilot period, with only one CHW in Rutana Province charging the device twice."- (*Bauler S. et al., 2025)<br>2. *"Other recommendations included finding a solution for the slow display of respiratory rate, portable backup battery packs to recharge the devices, and strategies for calming agitated children"* - (Bauler S. et al., 2025)<br>3. *"health workers were concerned that no device maintenance was scheduled and described issues related to device malfunction and charging (eg, lack of electricity, needing to monitor and charge the battery, device not being usable while charging"* - (Rawat A. et al., 2023)

Main (Overarching) theme	Categories	Device	Example quotes
	**Struggle with agitated child**	ChARM	1. *"Health workers also described increased consultation times using ChARM when the child was moving or agitated, severely malnourished, or fearful of the device, especially in pastoralist areas"* - (Rawat A. et al, 2023)<br>2. *"Distress was associated with seeing a new device, attaching ChARM to the abdomen, and sick children tended to be more distressed" - (Ward C et. al., 2019)*
		Masimo Rad-G	*1."Not very easy for the small and turbulent child, but for the child a little old, he could accept that we put the probe on him without problem" -* (Bauler S. et al., 2025)<br>2. "*Health workers recommended improving the functionality and sensitivity of Rad-G so the probe attachment could be easily used while children are moving or agitated."*- (Rawat A. et al., 2023)<br>3. "*There are also children who cry or react distressfully to the device"- (Rawat A. et al.,* 2023*)*<br>4. "*with the older infants I have struggled because they move a lot, cry a lot"* - (Sarine E. et al., 2021)<br>*5."*Agitation complicated measurements by the multimodal oximeter*"* - (Tack B. et al. 2022)<br>*6. "The device displays an 'error' message if the child moves or 'there is motion' and this 'distorts' the assessment, 'affects the result' and makes it difficult to 'get the correct result"*- (Baker K. et al., 2020)<br>*7. "They felt a lot of care was needed to control the motion of the child and to 'focus on the measurement' and follow the assessment process"* - (Baker K. et al., 2020)<br>*8. "Several HEWs talked more generally about children being distressed when they saw the device or the red light, but this was transient, and many calmed down when shown the pictures or the 'game' on the device screen"* - (Baker K. et al., 2020)
	**Feedback regarding device design**	ChARM	1. *"However, the red and green indicators were found helpful, and caregivers mentioned how the FCHV would show the red/green light to communicate the result of the assessment." - (*Källander K. et al., 2020)<br>2. *"More HEWs than FLHFWs expressed initial fear that the device was too complicated, difficult to operate" - (Ward C. et al., 2019)*

Main (Overarching) theme	Categories	Device	Example quotes
		Masimo Rad-G	1. *"And when I saw it for the first time, inside, there was a light that looked like fire; we thought it could burn the child"* - (Bauler S. et al., 2025)<br>2. *"However, the CHWs noted that the probes were more difficult to keep safe, as there was no mechanism to attach them to the device safely (they were left dangling)"* - (Bauler S. et al.,2025)<br>3. *"But there are questions that cannot be answered without knowing threshold values in order to say this or that another suffers from this disease." - (*Bauler S. et al., 2025)<br>4. *"Rad-G does measure pulse rate, but health workers were not trained on this as part of the ARIDA initiative]"*- (Rawat A. et al., 2023)<br>5. *"Another problem with the interface related to selecting age categories; some FLHFWs explained that the categories are too close to each other resulting in other categories 'being tapped' by mistake"* - (Baker K. et al., 2020)<br>6. *"Many HEWs and FLHFWs expressed no difficulty in reading the oxygen and RR levels and that the display was 'readable', 'clear' and the colours helped to interpret positive and negative readings"* - (Baker K. et al., 2020)<br>7. *"End-users were in favor of the size and weight of the multimodal oximeter"* - (Tack B. et al. 2022)<br>8. "The interviewed staff further reported that the alarm sounds of the multimodal oximeter worsened agitation of the child and alerted the children's caretakers who associated the oximeter with severe illness and poor prognosis" - (Tack B. et al. 2022)<br>9. *"Easy cleaning, rechargeable with USB cable" -* (Tack B. et al. 2022)
**Theme 3:** Perceived Acceptability and impact	**Initial hesitance and eventual trust**	ChARM	1. *"Though there was an initial hesitancy, the majority opined the device was user-friendly and could be used after proper training was imparted."* – (Ghosh N. et al., 2022)<br>2. *"Many FCHVs were initially concerned about their ability to use ChARM, and several mentioned that they found it 'really difficult' to learn how to use it, that they felt 'uncomfortable during the training' or they were 'intimidated' by the device. "However, for most, this initial reaction was short-lived, and FCHVs said they got more comfortable using it with time and practice." - (*Källander K. et al., 2020)

Main (Overarching) theme	Categories	Device	Example quotes
			3. *“Their impression changed and confidence grew after they used the device in practice” - (Ward C et al., 2019)*
		Masimo Rad-G	1. *“The children were scared at first. They thought we were going to stick them with needles (vaccination) like we used to do for the thick drop” - (Bauler S et al., 2025)*<br>2. *“However, FGD participants noted that most children calmed down once the probe was placed on the child’s finger” - (Bauler S. et al., 2025)*<br>3. *“Caregivers also perceived the device as a new technology that could help frontline health works identify diseases more accurately and quickly” - (Bauler S. et al., 2025)*<br>4. *“Even if children were crying before diagnosis, they get calm and relax when they see the device” - (Rawat A. et al.,* 2023*)*<br>5. *“Another thing is that people have come to know that there is this machine. They feel happy. It is enough that there is this machine.”*- (Kumar H. et al.,2021)<br>6. *“Many HEWs and FLHFWs expressed initial hesitation or fear of the device, explaining how they expected it be ‘confusing’ or ‘difficult to operate’, that they ‘hesitated’ due to lack of knowledge about the device and how they ‘feared’”* - (Baker K. et al., 2020)<br>7. *“These initial concerns were alleviated with practice, and most health workers said their skills, knowledge and ability to use the device had improved with time and experience”* - (Baker K. et al., 2020)
	**Impact of the device**	ChaRM	1. *“FCHVs were of the view that caregivers trusted them” - (*Källander K. et al., 2020)<br>2. *“HEWs desired to use the device in future because of the burden of pneumonia in their community and ‘number of affected children” - (Ward C et al., 2019)*
		Masimo Rad-G	1. *“We do not have to count respiratory movements manually as before as the method brings bias measurement” - (*Bauler S.et al., 2025)<br>2. *“The majority of health workers felt caregivers reacted positively to the devices and often requested they use the devices since becoming aware of them from HEWs or through community engagement” - (*Rawat A. et al., 2023)

Main (Overarching) theme	Categories	Device	Example quotes
			3. *"There was consensus among FLHFWs and HEWs, that they preferred Rad-G compared with previous manual counting methods"* - (Baker K. et al., 2020)<br>4. *"Caregivers' preferred for assessment with the device, and their belief that their children recover quickly if assessed with a 'medical device like Rad-G rather than giving treatment without a device"* - (Baker K. et al., 2020)
